# A Microfluidic Platform Containing Sidewall Microgrooves for Cell Positioning and Trapping

**DOI:** 10.5772/60562

**Published:** 2015-01-01

**Authors:** Masoud Khabiry, Nader Jalili

**Affiliations:** 1 Piezoactive Systems Laboratory, Department of Mechanical and Industrial Engineering, Northeastern University, Boston, USA; 2 Bioengineering Department, Northeastern University, Boston, USA

**Keywords:** Microfluidic, Cell Positioning and Trapping, Microcantilever-based Biosensors

## Abstract

Microfluidic channels enable the control of cell positioning and the capturing of cells for high-throughput screening and other cellular applications. In this paper, a simple microfluidic platform is proposed for capturing small volumes of cells using sidewall microgrooves. The cell docking patterns in the channels containing sidewall microgroove are also studied. Both numerical and experimental investigations are performed within channels containing sidewall microgrooves of three different widths (i.e., 50, 100 and 200 μm). It is observed that channels containing sidewall microgrooves play an important role in regulating cell positioning and patterning. The obtained results revealed that 10 to 14 cells were positioned inside the sidewall channels of 200 μm width, two to five cells were positioned within the channels of 100 μm width, and one to two individual cells were docked within the sidewall channel of 50 μm width. Particle modelling shows the prediction of cell positioning within sidewall microgrooves. The positions of cells docked within microgroove-containing channels were also quantified. Furthermore, the shear stress variation and cell positioning in the sidewall microgrooves were correlated. Therefore, these sidewall microgroove-containing channels could be potentially useful for regulating cell positioning and patterning on two-dimensional surfaces, three-dimensional microenvironments and high-throughput screening. Cell patterning and positioning are of great importance in many biological applications, such as drug screening and cell-based biosensing.

## 1. Introduction

Mcrofluidic platforms hold great promise for biochemical synthesis, high-throughput drug screening, and cell-based biological assay [1–5]. Microfluidic devices offer the possibility of controlling fluid flow, generating stable concentration gradients and regulating cell-soluble factor interaction in a temporal and spatial manner [6–9, 40]. The poly(dimethylsiloxane) (PDMS)-based microfluidic devices offer a number of advantages, such as low cost, short reaction time, high-throughput analysis, and realtime monitoring of biological processes [10–12, 38–40]. Furthermore, the microfluidic devices enable the control of cell docking and immobilization in a well-defined micro-environment, features necessary for cell-based screening applications [13–18]. Moreover, cell patterning and positioning are of great importance in many biological applications, such as drug screening and cell-based biosensing [39]. It has been shown that microfluidic devices containing shear-protective microgrooved regions located at the bottom of the substrate have the ability to control cell positioning [15–16]. The previous microgrooved regions located at the bottom of the channels provided shear-protective regions and regulated micro-circulation, resulting in cell docking and positioning; however, these approaches have some limitations, such as the fact that cells were attached to the bottom substrates so that it might be difficult to control the docking of a small number of cells. To overcome these challenges, we consider sidewall microgroove-containing channels to regulate cell docking and positioning. By using the sidewall microgrooves in the microchannels, we enable the capture of a small number of cells within the microfluidic device, showing more control over cell docking and positioning. Furthermore, it may be possible to co-culture different cell types in the sidewall microgrooved channels. It has been shown that a microfluidic system containing high-quality, small volumes of cells is required for studying quantitative system biology [19–20]. For example, a cup-shaped, high-density hydrodynamic cell isolation microfluidic device has been previously developed [19]. Individual cells were docked within cup-shaped microstructures and single-cell enzymatic kinetics was analysed. Two-layer cup-shaped arrays allow for the fluidic streamlines necessary for the cell trapping. When one cell was occupied within a cup-shaped array, the flow was diverted and then another cell was trapped within a neighbouring cup-shaped array. Furthermore, a microfluidic cell pairing device has been developed to study electrical fusion analysis [20]. Two cell types were captured and paired in two cup-shaped cell isolation microfluidic devices containing a larger capture cup and a smaller back-side capture cup. Although this microfluidic channel enables the capture of individual single cells within both the larger front-side cup and the smaller back-side cup, a complex three-step cell loading is required. Moreover, a multilayer microfluidic device with permeable polymer barriers for the capture and transport of cells with micro-valves was developed, which requires alignment and a complex fabrication process [41]. In contrast, our proposed microfluidic platform provides significant advantages over these methods. (i) It is very simple; (ii) it is one-layer; (iii) it provides a platform for capturing a small number of cells; (iv) it uses a one-step microfabrication process which does not require any alignment between the bottom substrate and the microfluidic channel; and (v) it allows for high-density microscopic analysis.

In this paper, a microfluidic device containing sidewall microgrooves that enables the trapping and positioning of cells in a controlled manner is developed. Furthermore, cell positioning in sidewall microgrooves is analysed. The effect of the cell docking and positioning on the sidewall microgroove-containing channels is also investigated. Computational simulations provided estimates of particle tracing patterns, which were accurate proxies for cell positioning. Computational modelling is compared to the experimental results of cell docking within the sidewall microgrooved channels. The particle trajectory was also predicted in sidewall microgrooves containing square microgeometry. Both numerical and experimental results are presented to demonstrate that the proposed microfluidic device containing sidewall grooves in the microchannel could be a potentially useful tool for studying the docking and positioning of small numbers of cells, down to one to two individual cells.

## 2. Materials and Methods

### 2.1 Fabrication of the microfluidic device containing sidewall microgrooves

**Figure 1.**
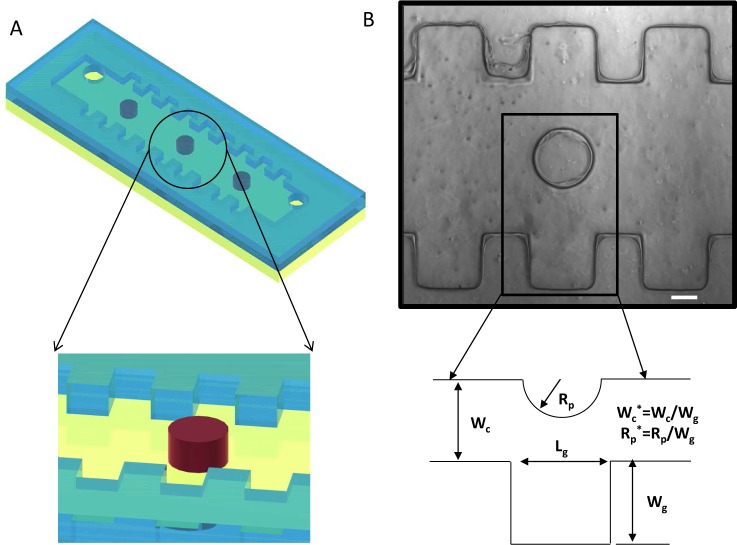
Microfluidic channel with sidewall grooves. (A) Schematic of the microfluidic channel containing the side wall grooves; (B) Phase contrast image of sidewall grooves in a microchannel (top view, scale bar:100 μm) and illustration of geometric dimensions of the device: W_c_ half of channel width, R_p_ post radius, L_g_ groove length, W_g_ groove width.

Microfluidic devices with sidewall microgrooves were fabricated using the photolithography technique that has been previously developed [21–24] (Figure 1). The silicon master mould was made using a negative photoresist (SU-8 2050, Microchem, MA). To make sidewall microgroove patterns of 80 μm thickness, SU-8 2050 was spin-coated at 1,500 rpm for 60 sec, baked for 8 min and 25 min at 65 °C and 95 °C, respectively, and exposed to UV for 3 min. After UV exposure, the photoresist-patterned silicon master was post-baked for 1 min and 8 min at 65 °C and 95 °C, respectively. The negative replica of the microfluidic channel was moulded in poly (dimethylsiloxane) (PDMS) (Sylgard 184 Silicon elastomer, Dow Corning, MI). The PDMS prepolymer mixed with silicone elastomer and curing agent (10:1) was poured on the master and cured at 70 °C for two hours. PDMS moulds were removed from the photoresist-patterned master. An inlet and outlet of the microfluidic channel were punched by sharp punchers for cell seeding and medium perfusion. The sidewall microgroove-containing channel and the bottom PDMS substrate were irreversibly bonded using oxygen plasma (5 min at 30 W, Harrick Scientific, NY). Sidewall microgrooves in the microchannels were placed perpendicular to the fluidic flow direction in the microfluidic device.

### 2.2 Cell docking in a microfluidic device

**Figure 2.**
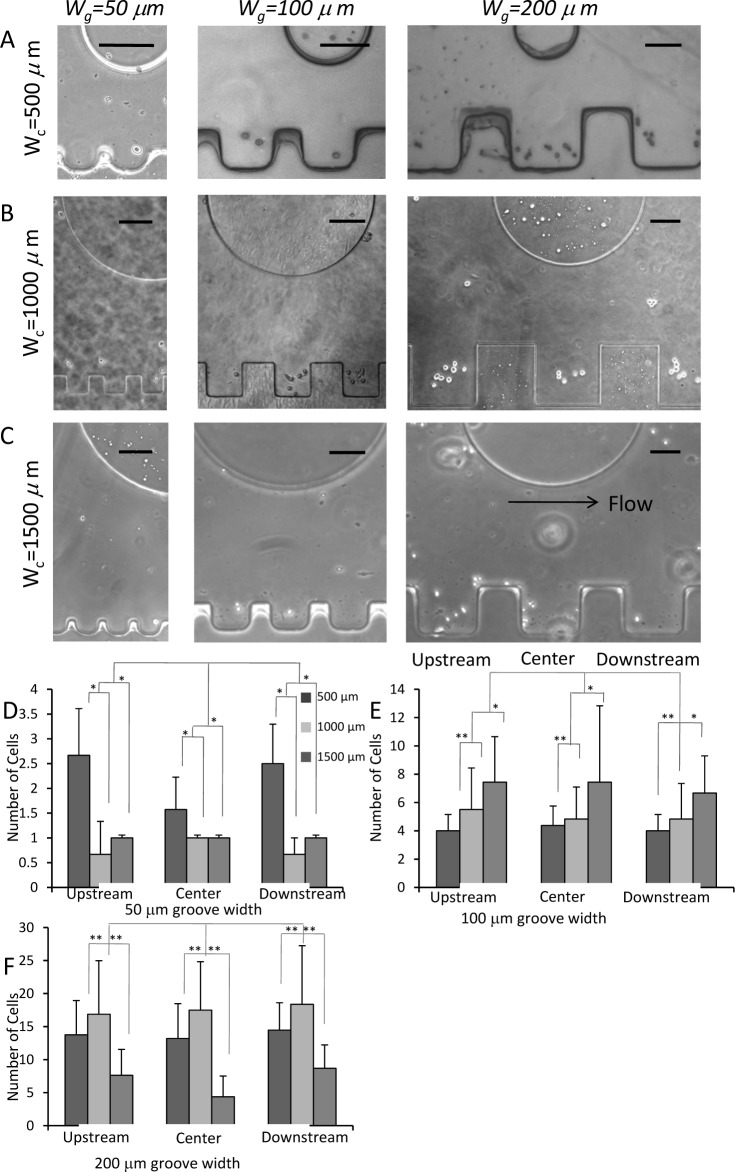
Cell docking in sidewall grooves. (A-C) Phase contrast images of cell docking within sidewall grooves (W_g_=50, 100, and 200 μm) in a microchannel (W_c_=500, 1000, and 1500 μm). Scale bars: 100 μm. (D-F) Quantitative analysis of cell docking within sidewall grooves (W_g_=50,100, and 200 μm) at upstreatm, center and downstream in a microchannel (W_c_=500, 1000, and 1500 μm).

NIH 3T3 mouse fibroblasts were cultured in Dulbecco's Modified Eagle Medium (DMEM, Invitrogen, CA) containing 10 % fetal bovine serum (FBS, Invitrogen) and 1 % penicillin/streptomycin (Invitrogen, CA). To seed the cells into the microfluidic sidewall channel, the cells were trypsinized and dissociated with culture medium. A counting chamber, also known as a hemocytometer, was used to obtain the cell density. The cells were seeded in a microfluidic device through a cell inlet port at the cell density of 6×10^6^ cells/mL. After 20 min cell seeding, the medium was infused using a syringe pump at a flow rate of 5 μL/min. The medium was pumped to the inlet port of the microfluidic device (the obtained flow direction was from left to right, as illustrated by an arrow in Figure 2C). We analysed cell docking in the sidewall microgroove-containing channels with three different widths of sidewall microgrooves (i.e., 50, 100 and 200 μm widths).

### 2.3 Image analysis for cell docking and retention

Cell images were obtained using an inverted microscope (Nikon TE 2000-U, USA). To analyse cell docking within sidewall microgrooves in the microchannel, we obtained cell numbers and their location through image analysis. The average cell size in the microgrooves was quantified by ImageJ software. The size of the loaded 3T3 fibroblast cells was on average 10 μm. The experiments were performed with different microgroove sizes three times in a microfluidic device. Statistical analysis was performed using the student t-test.

### 2.4 Numerical simulations

Computational fluid dynamics (CFD) was used to simulate the fluid flow behaviour in the sidewall microgroove-containing channels using the finite element method (COMSOL 3.4, Burlington, MA). There exists ample literature on CFD, finite volume, finite element methods [25–27] and fluid flow in cavities [28–35].

To estimate the cell penetration into the sidewall microgrooves, we performed a numerical simulation of our experimental setup. In our modelling, the unstructured mesh generation method was used for constructing the 3D mesh domain. Our fluid modelling is based on incompressible Navier-Stokes equations [36, 37] with the Stokes hypothesis assumed in conservation form for an arbitrary geometry. The governing equations can be written as follows:

Continuity equation:

(1) $$\frac{\partial \rho }{\partial t}+\vec{\nabla }\left(\rho \vec{V}\right)=0$$

Momentum equation:

(2) $$\rho \left(\frac{\partial \vec{V}}{\partial t}+\vec{V}•\vec{\nabla }\vec{V}\right)=-\vec{\nabla }p+\mu \nabla^{2}\vec{V}$$

where $\vec{V}$ represents velocity (m/s), p pressure (Pa), ρ density (kg/m^3^), μ dynamic viscosity of fluid (Pa Sec), and *t* time (sec). The properties of fluid (medium) in our modelling are considered to be the same as those of water; which implies the density of 1000 (kg/m^3^) and dynamic viscosity of 0.001 (Pa Sec).

For our numerical modelling, the boundary conditions at the walls and at the bottom of the microgrooves is set as no-slip boundary conditions. The specified velocity condition is applied for the inflow boundary condition (Dirichlet boundary condition). Moreover, the specified pressure is used for the outflow boundary condition (Dirichlet boundary condition). The outlet static pressure of 0 (Pa) is applied for our case. Furthermore, the criteria for convergence (RMS residual) are considered to be equal to 10^−6^.

## 3. Results and Discussions

### 3.1 Sidewall microgroove-containing channels

A PDMS-based microfluidic device with sidewall microgrooves was developed to regulate and control cell positioning and docking (Figure 1). This microfluidic device mainly consists of sidewall microgrooves (50×50, 100×100 and 200×200 μm) and posts (125, 250 and 375 μm radius) that enables control of flow velocity and shear stress profiles. Three types of microchannels (500, 1000 and 1500 μm widths) were fabricated. A microchannel with a 500 μm width has a 250 μm post diameter; a microchannel with a 1000 μm width has a 500 μm post diameter; and a microchannel with a 1500 μm width has a 750 μm post diameter. As shown in Figure 1B, our fluidic channel containing sidewall microgrooves was irreversibly bonded to a PDMS substrate. To analyse cell positioning within sidewall microgrooves, cells were seeded into a microfluidic device through a cell inlet port and medium was subsequently infused using a syringe pump. This microfluidic device has several advantages over previous cell docking microfluidic platforms, because we can regulate the docking of small numbers of cells, down to one to two individual cells, fewer than in the earlier studies [15–16]. The one-step microfabrication process we used in this paper did not require any alignment between microgrooves and the microfluidic channel layer, whereas it is an essential part of other approaches [19, 41].

Sidewall microgrooves were fabricated in the channels to analyse cell docking behaviour without any of the gravity effect which is usually generated within bottom-micro-grooved channels [15–16]. It is shown that the cell docking in bottom-microgrooved channels is significantly regulated by the gravity and shear stress profiles [15–16]. Thus, it is not easy to identify which parameter is more important to regulate and control cell docking and positioning. To address this issue, sidewall microgrooves were developed in the microchannels. Additionally, to better understand the effect of geometrical factors, four different parameters were varied, which include post radius (R_p_: 125, 250, 375 μm), channel width (W_c_: 500, 1000, 1500 μm), microgroove length (L_g_: 50, 100, 200 μm), and microgroove depth (W_g_: 50, 100, 200 μm). The geometrical parameters involved are shown in Figure 1B. These four spatial variables were also scaled by the microgroove width W_g_ which is equal to the microgroove length L_g_ in our studies. Moreover, two dimensionless ratios were defined W_c_*=W_c_/W_g_, and R_p_*=R_p_/ W_g_. As a result, the number of geometrical factors involved was reduced to two dimensionless ratios. The obtained values of R_p_* corresponding to the microfabricated microfluidic devices are 0.625, 1.25 and 1.875, respectively. We experimentally and theoretically evaluated the effect of these different parameters for cell docking and positioning within sidewall microgroove-containing channels.

### 3.2 Cell positioning within sidewall microgrooves

Cell docking and positioning were analysed within a microfluidic channel containing sidewall microgrooves (50, 100 and 200 μm widths) (Figure 2). The distance between the centres of one microgroove to the next one is equal to 1.5 W_g_.

Figure 2A-C represents the cell distribution within the sidewall microgrooves. Through an image analysis approach, the number of cells and their position within sidewall microgroove channels were obtained. As discussed earlier, the flow direction is presented in Figure 2C by an arrow, and is from left to right. Hence, the centre, upstream and downstream of the sidewall microgrooves are classified based on the flow direction and position of the post. Cell docking analysis showed that cell docking was significantly regulated by the geometry (i.e., groove width) of sidewall microgrooves. Figure 2D, E, F provides an easy comparison of cell counts for three different sidewall microgrooves (50, 100 and 200 μm in widths). It was found that different numbers of cells were docked within three sidewall microgrooves. It was revealed that two to five cells were positioned within 100 μm wide sidewall microgrooves, while 10 to14 cells were docked within 200 μm wide sidewall microgrooves in a microfluidic device with a 500 μm channel width. However, only a few cells (one to two) were docked within 50 μm wide sidewall grooves.

Cell docking results demonstrated that the number of cells docking within larger sidewall microgrooves (200 μm in width) is much higher than that of cells docking within smaller sidewall microgrooves (50 μm in width). Significant differences between the number of cells docked in the microfluidic device with 500 μm and 1500 μm channel widths were not observed. This could be related to the small size of the sidewall groove relative to the width of the channel itself. Not much difference in cell docking among the sidewall microgrooves of upstream, centre and downstream was observed. This can be explained by the fact that the post is located far away from the sidewall microgrooves. If the distance between a post and sidewall microgroove was short, the number of cells docking at the centre of the sidewall grooves might be higher compared to the upstream and downstream of the sidewall microgrooves. To confirm this hypothesis, fluidic flow and shear stress profiles were simulated. The obtained results for simulation are discussed in the theoretical modelling section. Generally, it was observed that cells were positioned and located at the centre of the sidewall microgrooves. Therefore, the obtained result for the sidewall microgrooves should prove useful for co-culturing different cell types.

**Figure 3.**
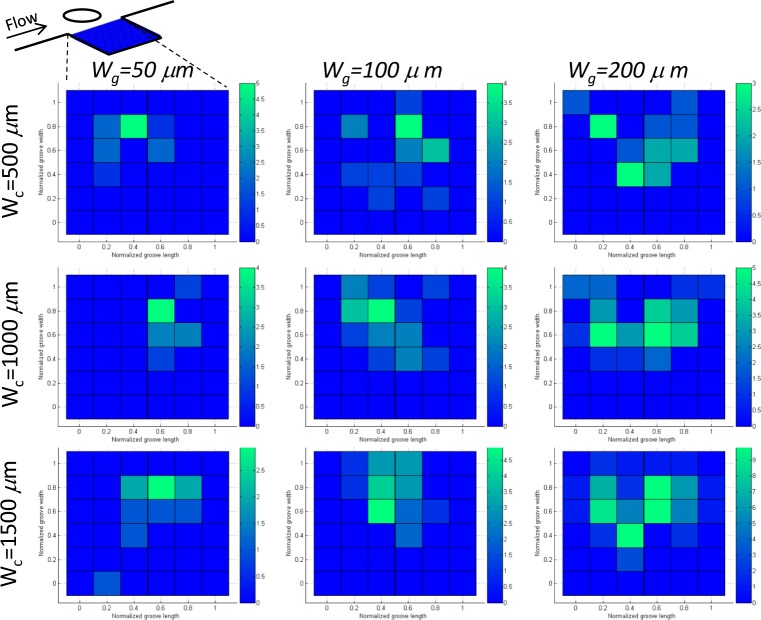
Cell distribution in sidewall grooves: Projection of 3D histogram in horizontal plane of cell distribution within side microgrooves (Wg=50, 100, and 200 μm) in a microchannel(Wc=500,1000, and 1500 μm);top left: illustration of the projected region in a sidewall groove.

To support our experimental data, cell docking and positioning were analysed using histograms. Figure 3 represents the two-dimensional projection of the 3D histogram for different microgroove sizes and channel widths. For comparison purposes, the length and width of all microgrooves for each channel width, 500, 1000 and 1500 μm, was normalized in Figure 3. The aforementioned histogram verifies that the distribution probability of cells inside the sidewall microgrooves is higher in the highlighted regions. It is also observed that the histogram distribution has a trend toward the central region of the sidewall microgrooves, and the probability of the cell docking is higher in the middle of the microgrooves. This observation indicates that cells will be docked within designated shear-protective sidewall microgrooves. Consequently, the cell docking and positioning can be regulated and controlled by this approach. It was noted that those cells that were not docked within the shear-protective region were removed by the medium perfusion.

### 3.3 Micropost design considerations

**Figure 4.**
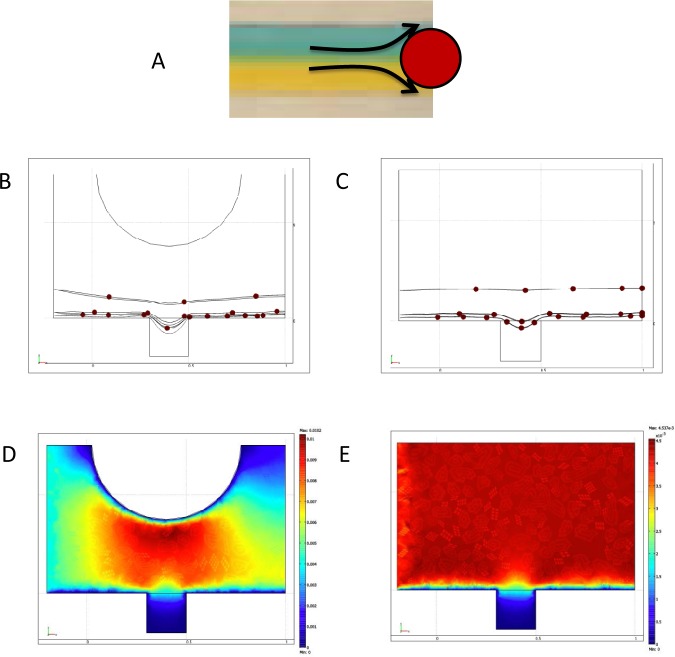
(A) Schematic presentation of flow diversion around a micropost (B,C) 3D particle simulation within the sidewall microgroove with micropost and without micropost. (D,E) Velocity distributions within the sidewall microgroove with micropost and without micropost.

**Figure 5.**
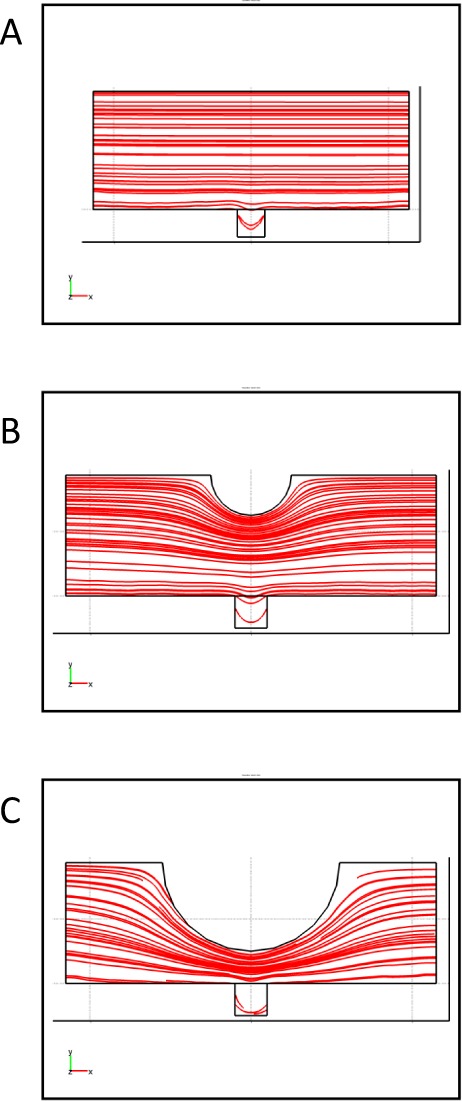
(A, B, C) Streamline distribution in the microchannel within the sidewall microgroove without micropost and with two different sizes of micropost

One of the distinct features of our microfluidic device relates to the incorporation of the microposts. These posts are aligned in the middle of the microchannels as shown in Figure 1A. The microposts play an important role in flow diversion. Moreover, these microposts facilitate the changing of the streamline patterns and velocity contours. A schematic presentation of flow diversion around the post is shown in Figure 4A. This diversion in the fluid flow should prove useful in case of delivering different drugs to the cells immobilized in the upper microgrooves as opposed to those residing in the lower microgrooves. The effect of the incorporation of the micropost on 3D particle simulation within the sidewall microgroove with and without micropost is illustrated in Figure 4B and C. In addition, the change in the velocity distributions within the sidewall microgroove with and without micropost is shown in Figure 4D and E. Furthermore, the effect of the inclusion of micropost and the effect of changing its diameter on the streamline distribution are demonstrated in Figure 5A, B and C. We note that by inclusion of the micropost the streamlines get closer together underneath the micropost. This streamline pattern change causes an increase in the fluid velocity below the micropost region while keeping the velocity in the microgroove area very low. Furthermore, through the particle simulation it was noted that inclusion of the micropost facilitates better particle penetration in the sidewall grooves.

### 3.4 Theoretical modelling of the cell position

A variety of numerical experiments for sidewall microgrooves were investigated. As mentioned earlier, to consider all the fabricated microfluidic devices, different geometry and channel sizes were simulated. Hence, three different microgroove sizes were considered. The series started from 200×200, continued to the size of 100×100, and concluded with the size of 50×50. The three-dimensional modelling of the sidewall microgrooves is considered since in our case studies the depth (perpendicular to the screen) to height ratio of the microgrooves was not above one. Therefore, it will not be justified to use two-dimensional modelling for the prediction of flow pattern and streamlines in our solution domains. In the modelling, the maximum Reynolds number was Re_*maxmax*_=0.375. This range of the Reynolds number is within the limit of laminar flow, or more precisely creeping flow, Re<1. Hence, the obtained experimental flow regime is in agreement with the presented numerical modelling and consistent with the assumptions made for analytical solution.

**Figure 6.**
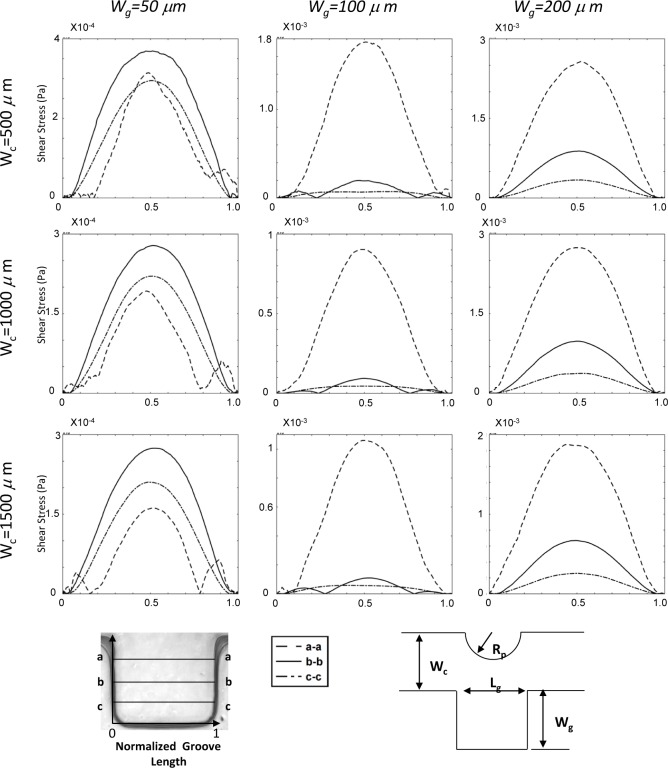
Shear stress distribution in sidewall microgrooves for three different geometry setup with (Wg=50, 100, and 200 μm) in a microchannel (Wc=500, 1000, and 1500 μm); bottom left: illustration of the specified paths which shear stress distribution shown along the horizontal lines a-a, b-b, and c-c.); bottom right: illustration of geometric dimensions

The shear stress variation inside the groove is shown in Figure 6. Different sections in the groove have been considered. The shear stress variation is shown for different groove sizes and different channel widths. In the groove itself, three horizontals (upper, middle and lower part) are shown by letters a, b and c (Figure 6). It was observed that the shear stress is one order of magnitude lower in the 50×50 groove size in comparison with 100×100 and 200×200 grooves. Moreover, there is not much variation from a-a, b-b and c-c sections of the 50×50 groove size. It was also noted that there are two more peaks in the shear stress profile (cc section) of the 50×50 groove size in comparison with the others. This observation can be explained by considering the fact that there is a combined corner and wall effect on this small region, and also the velocity is much lower in this region. In contrast, when the groove size becomes larger at 100×100 and 200×200, as shown in Figure 6, the shear stress becomes higher in the (a-a) section or upper part of the microgroove, whereas it decreases in the middle and especially the lower part of the groove. This explains why most of the cells accumulate in the middle section of the groove. It can be concluded that there is a threshold window where the cells prefer to stay in that region. It can also be seen that the experimental observations are in good agreement with the numerical simulations. The cells lie in the region predicted by the numerical modelling and can be correlated to the histogram of cell positions provided in Figure 3.

**Figure 7.**
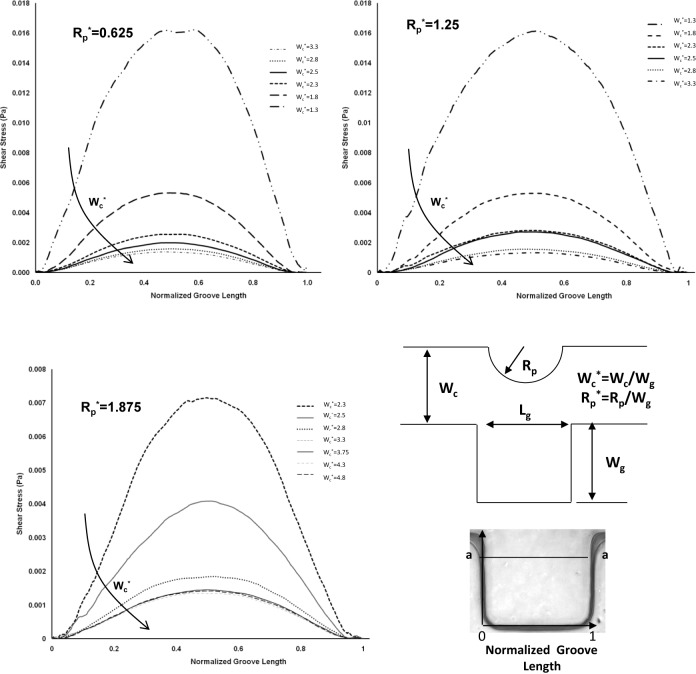
Shear stress distribution in sidewall microgrooves. (A) Effect of variation of channel width for three different pole radius R_p_*=0.625, 1.25, 1.875 along the horizontal line a-a (The schematic of the specified horizontal path a-a, and geometric dimensions).

To study the effect of varying the channel width on the shear stress variation inside the microgroove, the micropost radius was kept constant and the width of the channel was changed. Simulations were run for the representative values of R_p_* (0.625, 1.25 and 1.875), which correspond to the microfabricated microfluidic devices. For each case study, the microchannel width W_c_* was chosen as between 1.25 to 5 to cover all the corresponding microfabricated geometries. The results obtained for the upper region of the microgroove (a-a section) are shown in Figure 7.

The shear stress magnitude decreases by increasing the channel width, and this is applicable for all three microfabricated channel widths. As expected, as the microchannel becomes wider, the velocity and shear stress values decrease when all the parameters are constant. Furthermore, the obtained numerical results correlate with the effect of micropost size in the shear stress distribution and cell positioning. Therefore, microposts can play a role as a geometrical control over the cell positioning in the sidewall microgrooves.

**Figure 8.**
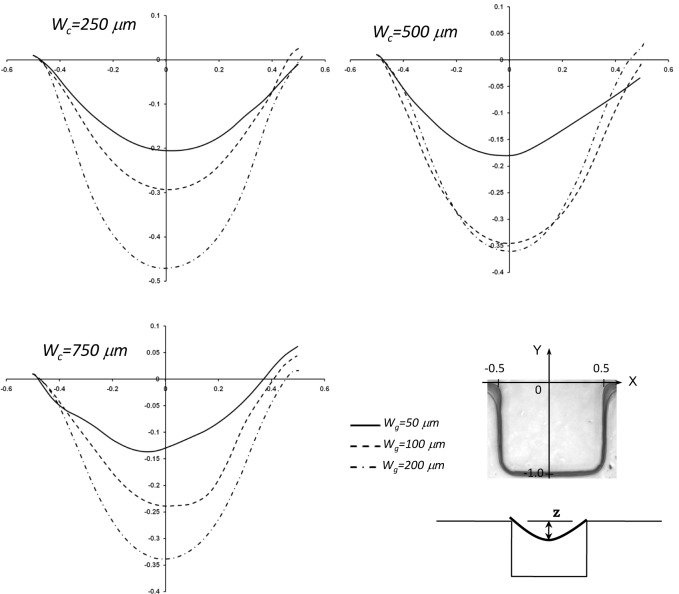
Cell penetration (z) in sidewall microgrooves for three different geometry setup with (Wg=50, 100, and 200 μm) in a microchannel (Wc=500, 1000, and 1500 μm); bottom right: illustration of the specified coordinates mapped in side wall groove and cell penetration (z)

To further understand the effect of variation of the width of microgroove on cell penetration, a set of numerical simulations was performed for three different geometry setups with a microchannel width of Wc (250, 500 and 750 μm). Cell penetration defined by Z is shown in Figure 8. In all cases we assumed that the micropost centre and the groove are aligned perfectly, and the microgroove lengths were normalized. Generally, it was noted that by increasing the microgroove size, the cell penetration is increased. These graphs are in agreement with the obtained values in the previous graphs and data.

## 4. Conclusions

In this study, we developed a unique and simple microfluidic platform for capturing a small volume of cells using sidewall microgroove-containing channels and microposts. It was demonstrated that the micropost size has an effect on the shear stress distribution inside the microgrooves. It was also observed that microgroove size plays a key role in cell capturing and cell positioning. In addition, the numerical modelling for predicting cell positioning inside the microgroove is presented. The effect of channel width variation on cell penetration is also investigated. Furthermore, the histograms of cell locations in the microgrooves were provided, and the most probable destination of the cells was shown. Sidewall microgroove-containing channels provide a platform for cell positioning and a shear-protected area for cell study, and are easily observable by a microscope. Hence, this simple yet adaptable microfluidic device should be useful for high-throughput screening, cell-based biological assay and cell-based biosensing, and also allow for high-density microscopic analysis with simplified image processing.

## 5. Supplementary Data

**Supplemental Figure 1.**
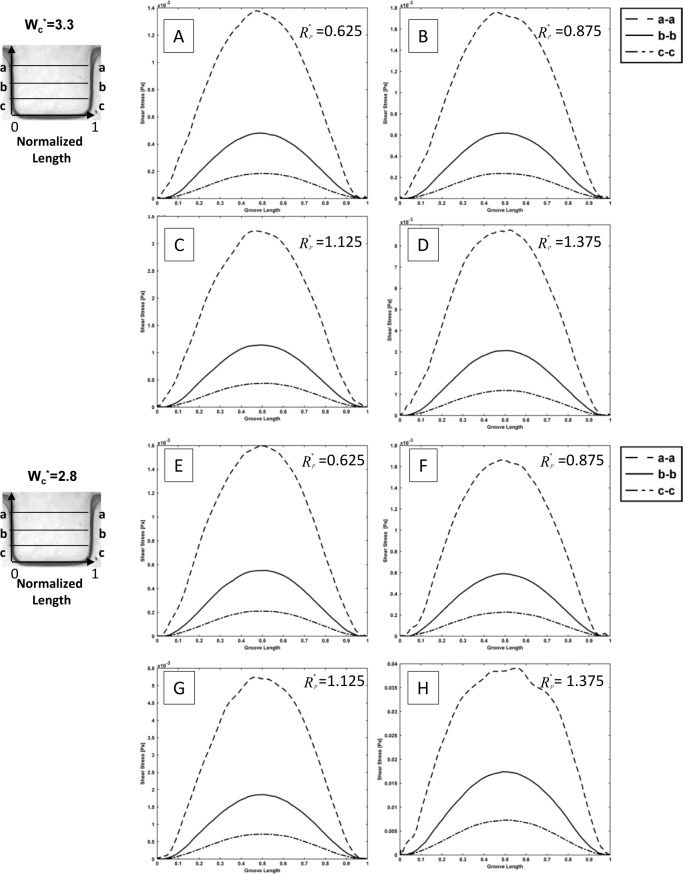
Shear stress distribution in sidewall microgrooves for different width and pole radius Wc*=3.3, 2.8, and R_p_*=0.625, 0.875, 1.125, and 1.375. The schematic of specified paths which shear stress distribution shown along the horizontal lines a-a, b-b, and c-c.

**Supplemental Figure 2.**
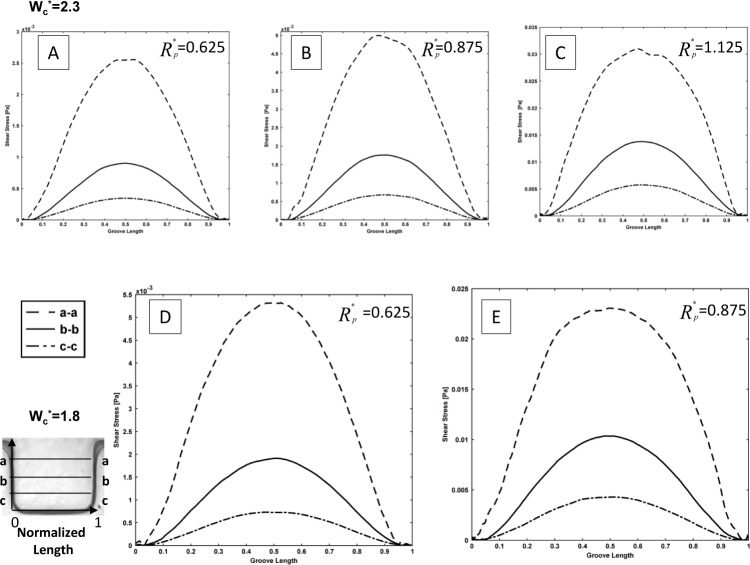
Shear stress distribution in sidewall microgrooves for different width and pole radius Wc*=2.3, 1.8, and Rp*=0.625, 0.875, and 1.125. The schematic of specified paths which shear stress distribution shown along the horizontal lines a-a, b-b, and c-c.

**Supplemental Figure 3.**
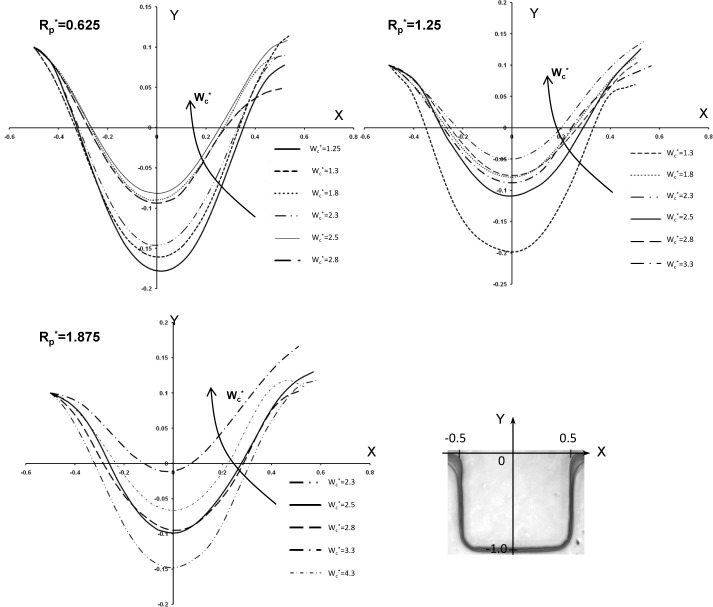
Effect of variation of width in the cell penetration for three different geometry setup with Rp*=0.625, 1.25, and 1.875 within the sidewall microgroove

To expand our numerical simulations, a variety of numerical experiments for sidewall microgrooves with dimensions other than those shown in Figure 6, 7 and 8 were investigated and the obtained results are presented in this section. The shear stress variation inside the microgroove is shown in supplemental Figures 1 and 2. Different sections in the groove have been considered and normalized by groove width (represented by the asterisk). The representative values of R_p_* (0.625, 0.875, 1.125 and 1.375) and microchannel width W_c_* (3.3, 2.8, 2.3 and 1.8) which were different to those of the experimental microfluidic devices were chosen. The shear stress distributions for these test cases are shown in supplemental Figures 1 and 2, respectively. It is observed from the supplemental Figures 1 and 2 that the shear stress is higher in the (a-a) section or the upper part of the microgroove, whereas it decreases in the middle (b-b) and especially the lower (c-c) part of the groove. This is consistent with the results obtained in Figures 6 and 7. Furthermore, the effect of variation of width on the cell penetration for three different geometry setups with R_p_*=0.625, 1.25 and 1.875 within the sidewall microgroove is presented in supplemental Figure 3. In these simulations, the values of the microchannel width W_c_* were varied from 1.25 to 4.3. It was observed that by keeping the micropost radius constant and increasing the microgroove width, the cell penetration is deepened. The obtained graphs reveal similar behaviour, as presented in Figure 8.
